# Bioluminescent Ross River Virus Allows Live Monitoring of Acute and Long-Term Alphaviral Infection by In Vivo Imaging

**DOI:** 10.3390/v11070584

**Published:** 2019-06-27

**Authors:** Essia Belarbi, Vincent Legros, Justine Basset, Philippe Desprès, Pierre Roques, Valérie Choumet

**Affiliations:** 1 Immunology of Viral Infections and Autoimmune Diseases, IDMIT Department, IBFJ, CEA, Université Paris Sud, INSERM U1184, 92265 Fontenay-aux-Roses, France; 2 Arbovirus group, Environment and Infectious Risks unit, Pasteur Institute, 75015 Paris, France; 3 Epidemiology and Physiopathology of Oncogenic Viruses Unit, Virology department, Pasteur Institute, 75015 Paris, France; 4 Université de la Réunion, INSERM U1187, CNRS UMR 9192, IRD UMR 249, Unité Mixte Processus Infectieux en Milieu Insulaire Tropical, Plateforme Technologique CYROI, 97491 Sainte Clotilde, La Réunion, France

**Keywords:** Ross river virus, alphavirus, in vivo imaging, viral persistence

## Abstract

Arboviruses like chikungunya and Ross River (RRV) are responsible for massive outbreaks of viral polyarthritis. There is no effective treatment or vaccine available against these viruses that induce prolonged and disabling arthritis. To explore the physiopathological mechanisms of alphaviral arthritis, we engineered a recombinant RRV expressing a NanoLuc reporter (RRV-NLuc), which exhibited high stability, near native replication kinetics and allowed real time monitoring of viral spread in an albino mouse strain. During the acute phase of the disease, we observed a high bioluminescent signal reflecting viral replication and dissemination in the infected mice. Using Bindarit, an anti-inflammatory drug that inhibits monocyte recruitment, we observed a reduction in viral dissemination demonstrating the important role of monocytes in the propagation of the virus and the adaptation of this model to the *in vivo* evaluation of treatment strategies. After resolution of the acute symptoms, we observed an increase in the bioluminescent signal in mice subjected to an immunosuppressive treatment 30 days post infection, thus showing active in vivo replication of remnant virus. We show here that this novel reporter virus is suitable to study the alphaviral disease up to the chronic phase, opening new perspectives for the evaluation of therapeutic interventions.

## 1. Introduction

Arthritogenic alphaviruses from the *Togaviridae* family such as Ross River virus (RRV) and chikungunya virus (CHIKV) are mosquito-transmitted viruses with positive-sense, single-stranded RNA genomes. These widely distributed arboviruses are responsible for severe musculoskeletal inflammatory diseases in humans [1,2]. RRV is endemic in Australia and Papua New Guinea and is responsible for 3 to 10 thousand cases per year. In 1979–1980, a large epidemic of RRV disease (RRVD) with 60,000 cases was reported in Australia and islands in the South Pacific [3]. In the beginning of 2017, the state of Victoria experienced an unusual outbreak of the disease, with a number of cases 20 times higher than previous outbreaks [4]. These epidemiological data illustrate the ability of these viruses to spread to naïve geographical regions with explosive outbreaks such as recently reported for CHIKV in the Caribbean and Latin America [5,6].

The clinical manifestations following infection with an arthritogenic alphavirus appear after a short incubation period (2–12 days) and usually include fever, maculopapular rash, myalgia and intense pain in the joints [3,7]. Approximately 10% to 30% of patients develop a chronic form of the disease with myalgia and poly-arthralgia persisting for months to years after the infection [3,8]. No specific therapies or licensed vaccines are currently available. The therapeutic management of patients is limited to supportive care with analgesics and anti-inflammatory drugs. The mechanisms underlying the persistent symptoms remain unclear, mainly due to the lack of adequate experimental models allowing their study. The first experimental animal models for alphaviral infection were developed in the 1970s, mostly using neonatal, young and immunocompromised mice. These animal models exhibited neurological and muscular damage but none of the disease key features such as arthritis and chronic manifestations [9,10]. Adult immunocompetent mice disease models were developed in 2006 for RRV and 2010 for CHIKV [11,12]; in these latest models, the infected mice presented symptoms similar to those seen in human cases, most importantly arthritis and bone loss. Non-human primates (NHP) models of chikungunya disease (CHIKVD) have also been developed and present disease manifestations very similar to those seen in humans [13]. While these models increased our understanding and gave new insights on the study of the acute phase of the disease, difficulties in monitoring the chronic manifestations makes them less suitable in studying the late stages of the arthralgic disease.

In vivo imaging of viral infection could help overcome the limitations of animal model studies. Indeed, the use of recombinant fluorescent and bioluminescent viruses allows non-invasive real-time imaging of viral replication in animals and constitutes a useful tool for pathogenesis studies [11,12,13,14]. Moreover, recent advances in the field gave the technique greater sensitivity and stability [15,16]. Several recombinant alphaviruses have been developed in the past. Sindbis virus, Western Equine and Venezuelan Equine Encephalitis viruses expressing the Firefly luciferase gave new insights in the physiopathology and dissemination of encephalitic infections in murine models [17,18,19]. When looking at arthritogenic alphaviruses, most studies focused on CHIKV with Renilla or Firefly luciferase reporters inserted under the control of a double subgenomic promoter. Notably, Ziegler et al. observed a bioluminescent signal limited to the inoculated foot after infecting mice with a Renilla expressing CHIKV [20]. This pattern was probably due to in vivo attenuation of the recombinant virus and made this model unsuitable for dissemination studies. Using a Firefly expressing CHIKV, Teo et al. [21] observed bioluminescence in the whole mouse body with a long-term signal in the inoculated foot up to 60 dpi, however this signal was moderate and did not allow extensive comparative studies. Sun et al. [22] tested several types of constructions and reporters to evaluate the efficiency of different cloning strategies for alphaviruses. They demonstrated that the use of smaller luciferases inserted without addition of an extra-subgenomic promoter increased the stability of the resulting viruses and reduced significantly their attenuation in vivo.

The chronic manifestations of the alphaviral arthritis are poorly understood due to difficulties in observing and monitoring chronicity and persistent infection in animal models. It has been shown in vitro that RRV could persistently infect macrophages, muscle cells and synoviocytes [23,24,25]. Viral RNA and antigens have been detected in synovial biopsies from patients infected with RRV and muscle biopsies from patients infected with CHIKV [26,27]. Nevertheless, no virus was directly observed or isolated in the chronic stages of the disease. Thus, it remains uncertain if the chronic arthritis is due to a persistent viral infection of the joints and associated tissues or to a chronic pro-inflammatory environment as seen during rheumatoid arthritis [28,29].

To study the dynamics and pathogenesis of arthritogenic alphaviruses infection in living animals up to the chronic stage, we generated an RRV expressing NanoLuc (NLuc), a protein smaller and brighter than the luciferase proteins previously used in the design of reporter viruses. The reporter virus RRV-NLuc described here expresses high levels of luciferase and exhibits near-native replication kinetics *in vivo*. We used this virus to perform *in vivo* imaging and tracked the viral replication and dissemination of RRV in a previously described mouse model for RRVD [11]. Using RRV-NLuc, we evaluated in real-time and in living animals the effect of an anti-inflammatory treatment strategy with Bindarit, an inhibitor of monocyte recruitment reported to reduce symptoms in several animal models of cancer and immune diseases and currently evaluated in a phase II trials in type 2 diabetic nephropathy patients [30].

Overall our study established the use of this reporter virus as a novel and powerful tool to study the kinetics of viral dissemination and pathogenesis, virus-host interactions as well as therapeutic interventions for alphaviral diseases.

## 2. Materials and Methods

### 2.1. Design of the Recombinant RRV-NLuc

The full cDNA clone of RRV T48 strain (GenBank accession number GQ433359) was obtained from R. Kuhn’s laboratory [31]. In order to generate a recombinant RRV virus expressing NanoLuc, we used a strategy previously developed in our laboratory [32,33]. The NanoLuc gene (NLuc) was inserted in the non-structural open reading frame (ORF) of the viral genome, between the nsP3 and nsP4 genes, flanked by two nsP2 cleavage sites. Thus, NLuc is expressed in the non-structural polyprotein during viral replication and released after nsP2 cleavage. The full sequence of RRV-NLuc was submitted to GenBank.

**Figure 1:**
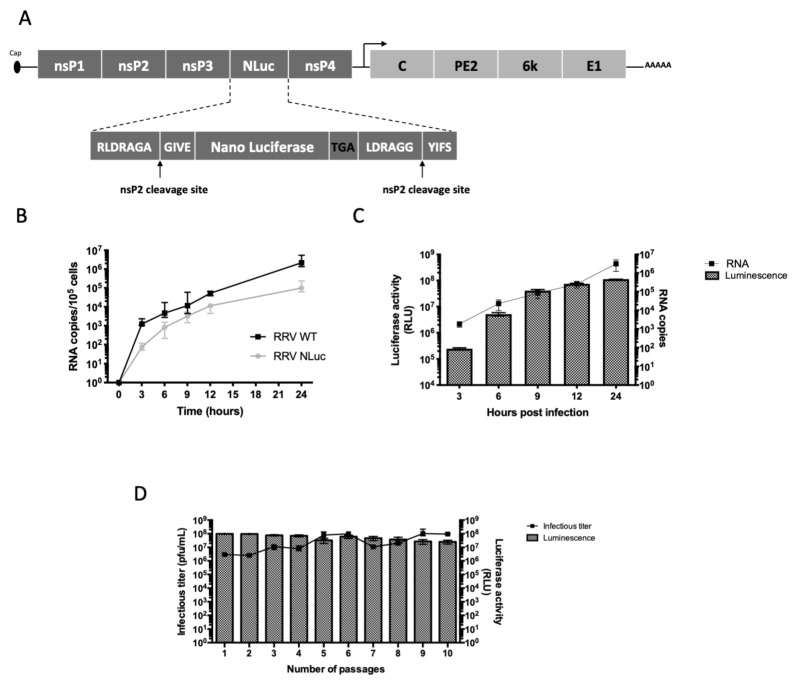
*In vitro* characteristics of the recombinant Ross River virus (RRV)-NanoLuc (NLuc). (**A**) Genome structure and site of the luciferase gene (Luc) insertion; (**B**) Replication kinetics of RRV-NLuc and the parental virus (RRV WT) on HeLa cells infected at a multiplicity of infection (MOI) of 1 C, total viral RNA was quantified at the different time points in cell-lysates, median and range are represented (Wilcoxon test, *p* > 0.05; Non-parametric Spearman correlation, *r* = 1, *p* = 0.002); (**C**) Luc activity and replication kinetics in HeLa cells infected with RRV-NLuc (MOI of 1), NLuc activity (grey bars) and intracellular viral RNA (black line) are shown (non-parametric Spearman correlation, *r* = 1; *p* = 0.01); (**D**) Stability of RRV-NLuc during 10 serial passages in vitro on HeLa cells (MOI of 1), samples were collected every 24 h, Luc activity expressed in relative light units (RLU), Infectious titer expressed in pfu/mL (non-parametric Spearman correlation test, *r* = 0.89; *p* = 0.001).

Briefly, we designed a synthetic sequence containing the C-terminal portion of the nsP3 gene followed by an nsP1/2 cleavage site, a first SpeI restriction site, the luciferase gene, a second SpeI site, the Opal stop codon of nsP3, the nsP3/4 cleavage site and finally the N-terminal part of the nsP4 gene (Figure 1a). This construct was inserted between the restriction sites SgrDI and ApaI of the RRV T48 molecular clone. We thus obtained an infectious clone of RRV expressing NanoLuc called pRRV-NLuc. The restriction enzymes used were from New England Biolabs (Ipswich, Massachusett, USA).

### 2.2. Production and Titration of the Viruses

The infectious cDNA clone was linearized by SacI (New England Biolabs) digestion, followed by phenol/chloroform extractions. The linearized templates were in vitro transcribed with mMESSAGE mMACHINE kit from ThermoFisher Scientific (Waltham, Massachusetts, USA); and the viral RNAs thus obtained were electroporated in BHK21 (ATCC CCL-10) cells. The viral supernatants were harvested after 48 h and used to infect new BHK21 cells and constitute a stock for the experiments. All the viral stocks produced were titrated by plaque assay on Vero cells [33].

### 2.3. In Vitro Replication Kinetics of the Recombinant and Parental Viruses

HeLa cells (ATCC CCL-2, LGC Standards S.a.r.l., Molsheim, France) were seeded at a concentration of 10^5^ cells/well in a 24-well plate and infected with RRV-NLuc or RRV WT at a multiplicity of infection (MOI) of 1. At 3, 6, 9, 12 and 24 hpi, the supernatant was removed, the cells were washed with phosphate buffered saline (PBS) and lysed with RA1 lysis buffer, RNA were extracted using the Nucleospin RNA II kit from Macherey Nagel (Berlin, Germany) according to manufacturer’s instructions. The extracted RNA was stored at −80 °C before quantification of viral RNA.

### 2.4. In Vitro Stability of the Recombinant Virus

HeLa cells (ATCC CCL-2) were seeded at a concentration of 10^5^ cells/well in a 24-well plate and infected with RRV-NLuc at a multiplicity of infection (MOI) of 1. At 24-hpi, 100 µL of the supernatant were used to infect fresh cells (dilution factor 1/10). The remaining supernatant was stored at −80 °C and later used for titration and the cells were washed with PBS and lysed with a passive lysis buffer from Promega, (Wisconsin, USA) and stored at −80 °C and tested for luciferase activity. The operation was repeated using the same dilution factor until passage 10, supernatants were then titrated by plaque assay and cell lysates were monitored for luciferase activity.

### 2.5. Luciferase Activity Quantification

Cells were lysed with a passive lysis buffer (Promega, Charbonnières-les-Bains, France) and clarified for 10 min at 13,000× *g*. Organs lysates were grinded and clarified for 10 min at 13,000× *g* then diluted at 1/10 in PBS. Luc activity was measured on a Centro LB 960 luminometer, Berthold Technologies GmbH (Bad Wildbad, Germany) by mixing 50 µL de the lysate sample with 50 µL of 1% furimazine substrate.

### 2.6. Viral RNA Extraction and Quantification

20 µL of blood were used for RNA extraction using the Nucleospin 96 RNA kit (Macherey Nagel) according to the manufacturer’s instructions.

Tissues samples collected and frozen at −80 °C, were weighed and grinded in Macherey Nagel RA1 Lysis buffer with a Precellys system^®^ and ceramic beads tubes, Bertin technologies (Montigny Le Bretonneux, France). Total RNA was extracted using Nucleospin RNA II kit (Macherey Nagel) according to manufacturer’s instructions.

Quantitative reverse transcriptase polymerase chain reactions (qRT-PCR) were performed in an Applied Biosystems 7500 fast thermocycler (ThermoFisher Scientific). The sequences of the primers (400 nM) and probe (200 nM) are the following:

RRV-F (position 10407): AGCAACAATCAGGATCAGTTAT;

RRV-R (position 10616): AATCTACCCGGCTGGCCTG;

RRV-Probe (position 10511): [FAM]TCTCAACAGCTTGGTCACCGTT [TAM].

qRT-PCR was performed on 5 µL of the extracted RNA with the following cycling conditions: 30 min at 56 °C, 5 min at 95 °C and 40 cycles at 95 °C for 15 s, 60 °C for 1 min.

### 2.7. In Vivo Stability of the Recombinant Virus

In order to assess the presence of the NLuc insert in vivo, the nsP3-nsP4 region of the viral genome was sequenced, cDNA was generated with SuperScript II from Invitrogen (California, USA) using the total RNA extracted from tissues at 6 dpi and the following primers: nsP3-fwd (5′-GTCTCTCCTACACCCACGCCTC-3′) and nsP4-rev (5′-GTTTCTCCCTGGCCTCATCC-3′). The cDNA was amplified by PCR using Phusion High-Fidelity DNA polymerase (Thermo Fischer Scientific). The PCR products were analyzed by agarose gel electrophoresis and ethidium bromide staining and purified using QIAquick PCR Purification Kit, Qiagen (Hilden, Germany) before Sanger sequencing (Eurofins Genomics, Luxembourg). The sequences obtained are available in GenBank under the accession numbers MH714854 to MH714857.

### 2.8. Ethics Statement

Mice were housed in the Institut Pasteur’s Animal facilities according to the French and European regulations on care and protection of Laboratory animals. This work was approved in 2014 by the Animal Ethics Committee of Pasteur Institute (CETEA n°89-Institut Pasteur) and by the French Ministry of Higher Education and Research (MESR 00762.02).

Anesthesia was performed using injectable ketamine/xylazine mix for infections and isoflurane inhalant anesthetic during imaging procedures. Euthanasia was performed by cervical dislocation or intra-cardiac phosphate buffered saline (PBS) perfusion after ketamine/xylazine anesthesia for tissues-collection.

### 2.9. Mouse Experiments

Three- to six-week-old albino C57BL/6N mice (B6N-*Tyr*^C-Brd^/BrdCrCrl) were purchased from Charles River Laboratories (Saint Germain Nuelles, France). This albino strain, presenting a mutation in the tyrosine gene (B6N-*Tyr*^C-Brd^/BrdCrCrl), was chosen to minimize the light absorption by the black fur of classic C57BL/6 mice. After anesthesia, mice were infected subcutaneously in the rear footpad with different doses of RRV-NLuc (10^3^ to 10^6^ pfu) or RRV wild type. Mice were monitored for weight, clinical signs and viremia. Blood collections were performed on the caudal vein with heparin. Swelling of the inoculated foot was assessed by measuring the height and width of the perimetatarsal area using a caliper (Mitutoyo, Japan). The swelling is expressed as a percentage and calculated as follows:

(1) $$\frac{W_{if}\times B_{if}}{W_{cf}\times B_{cf}}\;\times \;100,$$

where W: Width, B: Breadth, *if*: Inoculated foot and *cf*: Contralateral foot.

### 2.10. Tissue Collection for Ex Vivo Experiments

Mice were anesthetized with a mix of ketamine (Merial, Lyon, France) and xylazine (Bayer, Berlin, Germany) mix before PBS intra-cardiac perfusion. Organs were collected and kept frozen at −80 °C.

### 2.11. Cyclophosphamide Treatment

For in vivo viral reactivation experiments, mice were injected intraperitoneally at 30 dpi with two doses of 150 mg/kg of cyclophosphamide from Sigma-Aldrich (Darmstad, Germany) in PBS at six days interval [34]. Vehicle treated mice received diluent alone. To monitor the immunosuppression, peripheral blood leucocytes counts were performed using a “Scil Vet abc” hematometer (Scil animal care company, Altorf, France) before the treatment and then every two days over 12 days.

### 2.12. Bindarit Treatment

Mice were injected intraperitoneally twice per day with a dose of 100 mg/kg of Bindarit from Euromedex (Souffelweyersheim, France) in PBS [35]. Vehicle treated mice received diluent alone. Treatment started 6 h prior to infection and lasted for five days.

### 2.13. Bioluminescence Imaging

Infected and mock-inoculated mice were intraperitoneally injected with furimazine (kindly provided by the Chemistry and Biocatalysis unit, Medicinal Chemistry group, Pasteur Institute, Paris) at a dose of 4 mg/kg of body weight and anesthetized with isoflurane for 5 min. In vivo images were acquired with an IVIS spectrum and IVIS spectrum CT systems (Perkin Elmer, Villebon-sur-Yvette, France) and analyzed with Living Image 4.5 software (Perkin Elmer). To quantify the bioluminescence, regions of interest (ROI) of the same surface were defined manually, and the data were expressed as total flux (number of photons per second (p/s)) or as radiance unit (p/s/cm2/sr: number of photons per second that leave a square centimeter of tissue and radiate into a solid angle of one steradian (sr)).

### 2.14. Statistical Analyses

Data are presented as mean ± SD. Statistical analyses were performed using GraphPad prism 6. Differences between groups and controls were analyzed using two way analysis of variance (ANOVA) followed by a Bonferroni post-test, Mann-Whitney and Kruskal-Wallis followed by Dune’s post-test. *p*-values inferior to 0.05 were considered significant. Boxplots show the median (central horizontal line) with 50% of the data centered on it. The vertical lines represent the 10th and the 90th percentile.

## 3. Results

### 3.1. Design of Recombinant RRV-NLuc and In Vivo Infections

In order to study the physiopathology of alphaviral arthritis, we engineered a new recombinant virus, RRV-NLuc (GenBank accession number MH714857), with a NanoLuc gene inserted in the non-structural region of the RRV T48 infectious clone’s genome between the nsP3 and nsP4 genes (Figure 1a). To examine their replication kinetics, we infected HeLa cells with equal MOIs of recombinant or parental virus. Despite a lower replication rate, RRV-NLuc exhibited replication kinetics similar to the parental virus (Figure 1b and Figure S5). We observed no significant differences in the intracellular viral RNA levels (Wilcoxon test, *p* > 0.05) and a correlation in the replication curves of the two viruses; (Non-parametric Spearman test, *r* = 1, *p* = 0.002). To assess the reporter gene expression, we measured the luciferase activity in cellular lysates at different times post-infection (Figure 1c). The bioluminescent signal was detected from 3 h post-infection (hpi) and reached a plateau at 24 hpi. To determine the stability of RRV-NLuc, we performed serial passages of the virus on HeLa cells. The luciferase activity and the infectious titer correlated up to 10 passages (non-parametric Spearman correlation test, *r* = 0.89; *p* = 0.001), demonstrating the stability of this construct and the reliability of the luciferase quantification as an indicator of viral replication (Figure 1d).

**Figure 2:**
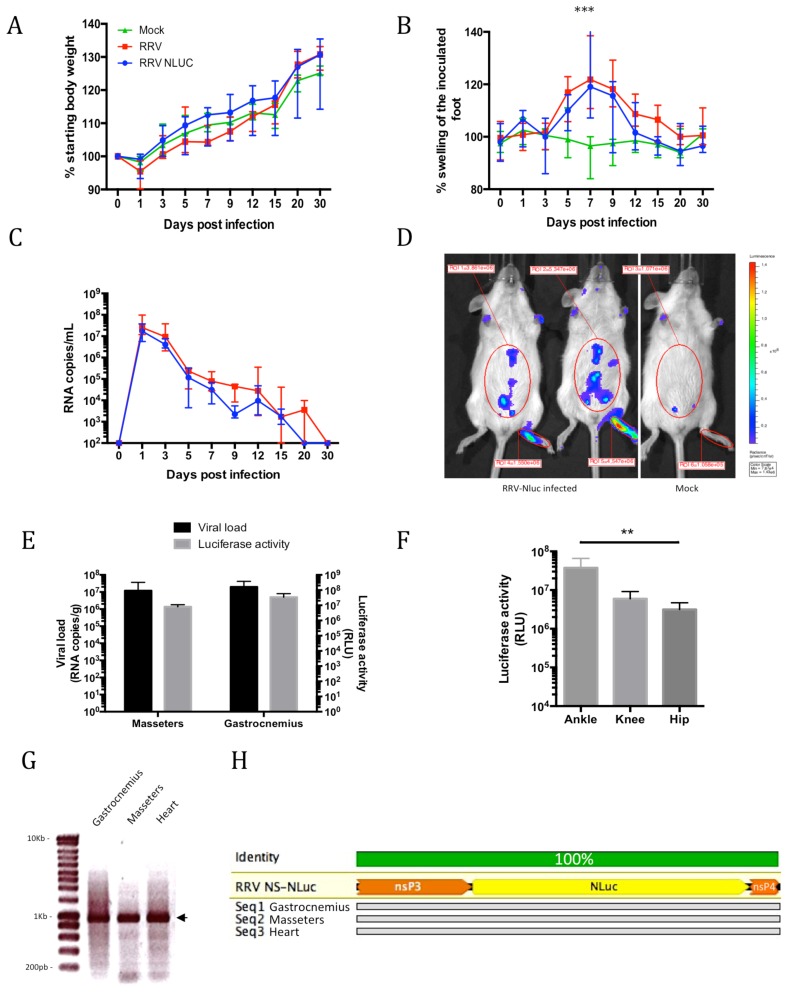
Experimental infections of immunocompetent albino mice with RRV-NLuc and the native RRV-T48. Mice were inoculated with 10^6^ pfu of RRV-NLuc (blue) or native RRV (red) or diluent alone (green), then monitored for the indicated parameters. Each data point is the mean ± SEM of four mice and is representative of two independent experiments. (**A**) Body weight gain (%); (**B**) swelling of the inoculated foot (*** = *p* < 0.001, two way ANOVA); (**C**) viremia in peripheral blood; (**D**) in vivo imaging of RRV-NLuc at 1 dpi, a high bioluminescent signal is observed in the inoculated foot; (**E**) assessment of viral load (RNA/g) and luciferase activity (RLU) in the masseters and gastrocnemius muscles at 6 dpi; (**F**) luciferase activity (RLU) in the joints of the inoculated limb (*p* = 0.005, Kruskal-Wallis); (**G**) electrophoresis of PCR products from total RNA extracted from gastrocnemius, masseter and cardiac muscles at 6 dpi. The amplified fragment, indicated by the arrow, contains the NLuc insert flanked by parts of nsP3 and nsP4 (expected size 950 bp); (**H**) alignment of the nsP3-nsP4 region with the NLuc insert (designed with Geneious software, Biomatters), the sequences shown were obtained 6 dpi from gastrocnemius, masseter and cardiac muscles, the insertion is present and no mutations were observed compared to the RRV-NLuc reference (data representative of three animals).

The insertion of a reporter gene in a viral genome can impact the replication and impair the development of the disease in vivo. To assess the in vivo virulence of RRV-Nluc, we infected albino immunocompetent mice with 10^6^ plaque-forming unit (pfu) of the wild type (WT) or recombinant virus. We monitored disease signs, weight and viremia during the acute phase of the disease (Figure 2). The recombinant RRV-NLuc and WT virus showed similar kinetics of replication *in vivo* and no difference was observed in weight gain between the two groups of animals (Figure 2a). The inoculation of arthritogenic alphaviruses in the footpad induces a swelling of the foot [12]. Both viruses induced a swelling of the inoculated foot, with a peak at seven days post-infection (dpi; *p* < 0.001), and no significant difference between the recombinant and native viruses (Figure 2b). For both viruses, the peak of blood viremia occurred at 1 dpi and slowly decreased, to reach undetectable levels at 25–30 dpi (Figure 2c). One day after inoculation of the recombinant virus, a significant bioluminescent signal could be detected in the inoculated foot and in the abdominal region, indicating active viral replication in these sites (Figure 2d).

RRV has a preferential tropism for muscles and joints tissues [10,11,36]. Viral replication in muscles has been described as early as 12 hpi and is active during the acute phase [11]. To assess the in vivo tropism of our novel recombinant virus, we collected gastrocnemius and masseters muscles at 6 dpi upon sacrifice. We observed high bioluminescent signal and viral RNA accumulation in these tissues (Figure 2e and Figure S1), thus indicating muscular tropism and reflecting the relationship between viral load and bioluminescent signal. To assess the replication of RRV-NLuc in the joints at 6 dpi, we collected the ankle, knee and hip joints from the inoculated limb and tested them ex vivo for the luciferase activity (Figure 2f). We observed the highest signal in the ankle joints, close to the inoculation site and a lower signal in the knee and hip joints indicating reduced viral replication in remote sites. Finally, to confirm the stability of the construct in vivo, we extracted total RNA from muscles at 6 dpi and amplified by reverse transcription PCR (RT PCR) the region of the NanoLuc insertion (Figure 2g,h). After electrophoresis of the PCR product, we observed bands at the expected size for all the tissues and mice tested (Figure 2g). Moreover, sequencing of the PCR products showed the absence of mutations in, upstream or downstream of the inserted gene (Figure 2h, GenBank accession numbers MH714854, MH714855 and MH714856). These data demonstrate the high in vivo stability of our NanoLuc construct and further confirm the reliability of the bioluminescent signal obtained.

### 3.2. Dynamics of the Acute Phase of Infection with RRV-NLuc

To characterize viral dissemination and tropism in vivo, we infected mice with low doses (10^3^ and 10^4^ pfu) or high doses (10^5^ and 10^6^ pfu) of RRV-NLuc. Disease signs, weight gain and viremia were monitored, and mice were imaged during the acute phase of RRVD.

**Figure 3:**
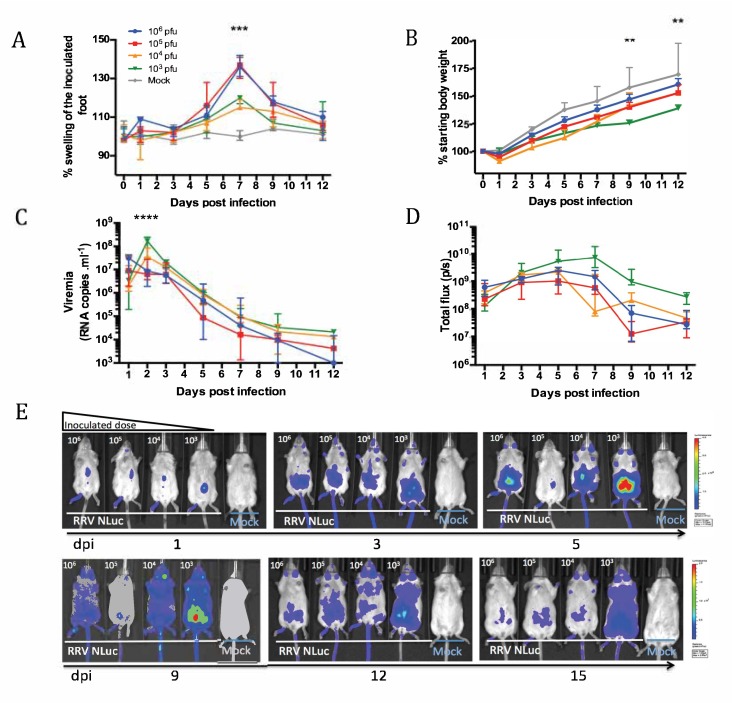
Dose-effect on the in vivo dissemination of RRV-NLuc during the acute phase. Mice were infected with doses ranging from 10^3^ to 10^6^ pfu of RRV-NLuc (10^6^ [blue]–10^5^ [red]–10^4^ [orange]–10^3^ [Yellow]-Mock [grey]). Mice were monitored for the indicated parameters. Each data point is the mean ± SEM of five mice and is representative of two independent experiments. (**A**) % Swelling of the inoculated foot; (**B**) body weight gain (%) significant difference observed at 9–12 dpi between the 10^3^ pfu group and all the other tested doses; (**C**) viremia in peripheral blood, significant difference observed at 2 dpi between the 10^3^ pfu group and all the other tested doses; (**D**) quantification of the bioluminescent signal (whole body) and (**E**) longitudinal imaging of the bioluminescent signal within five animals from 1 to 15 dpi. From the right to the left, decreasing inoculated doses and mock mice (10^6^–10^5^–10^4^–10^3^ - Mock), ventral side, scale upper panel (1–5 dpi) min = 5.10^6^ max= 4.10^8^ p/s/cm^2^/sr, scale lower panel (9–15 dpi) min = 1.10^5^ max = 2.10^7^ p/s/cm^2^/sr. (** = *p* < 0.01, *** = *p* < 0.001, **** = *p* < 0.0001, two way ANOVA).

We observed a swelling of the inoculated foot with all the tested doses with a peak at 7 dpi (*p* < 0.001). As expected, the severity of the swelling correlated to the inoculated dose (Figure 3a). However, the mice infected with 10^3^ pfu had the lowest weight gain compared to all the other tested doses (Figure 3a), reflecting the disease severity even at the lowest tested dose (9–12 dpi, *p* < 0.01).

As shown in Figure 3c, we observed a delayed infection when the mice were inoculated with the lower doses of viruses, with the peak in blood viremia observed at day one for the highest doses, and at day two for lower doses. Interestingly, the same trend was observed when monitoring the bioluminescent signal in the early days after the infection with a similar delay in the kinetics of accumulation of the virus in the whole mouse body as monitored by measurement of the bioluminescent signal (Figure 3d, 1–3 dpi).

In the RRV-NLuc infected animals, the bioluminescent signal was visible in the inoculated foot from day one (median signal 6.10^8^ photons/second (p/s)), increased until 5 dpi (median signal 2.10^9^ p/s) and decreased slowly from day seven on (*p* < 0.05, two way ANOVA; Figure 3e). From day one to day three, the bioluminescent signal correlated with the viremia (*p* = 0.0006 at 1 dpi; *p* = 0.03 at 3 dpi, Spearman correlation test). In the later stages of the infection, the bioluminescent signal reflected the invasion of mouse organs and accumulation of virus in the whole mouse body.

At 3 dpi, we observed a dissemination of the viral replication from the site of the inoculation in the foot to the whole limb, but also to the abdominal region and up to the masseter muscles as it was previously reported in mice for RRV [10]. Strikingly, the mice inoculated with the lowest doses of RRV-NLuc exhibited the highest and longest duration in bioluminescent signal from day three on (Figure 3d,e). We thus used this last inoculum dose (10^3^ pfu) in our experiments.

### 3.3. Role of Macrophages During Viral Dissemination in the Acute Phase

**Figure 4:**
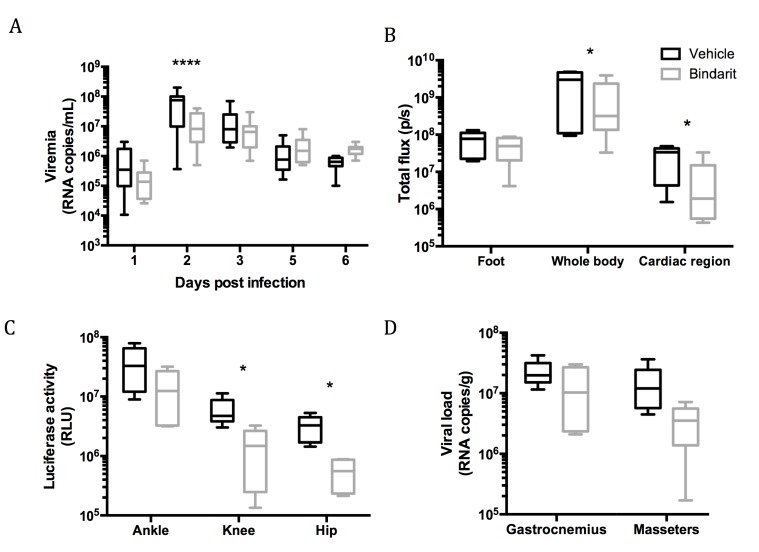
Bindarit effect on viral dissemination and replication. Mice were treated with Bindarit (grey) or vehicle treated (black) 6 h prior to infection with 10^3^ pfu of RRV-NLuc. Mice were monitored for the indicated parameters. Data were pooled from two independent experiments (*n* = 8). (**A**) Viremia in peripheral blood; (**B**) in vivo imaging at 5 dpi, quantification of the bioluminescent signal in the inoculated foot, cardiac region and whole body of the infected mice; (**C**) luciferase activity in the joints of the inoculated limb at 6 dpi (mean background signal 1.7 10^4^ RLU) and (**D**) RNA loads in muscles at 6 dpi (*n* = 5). * = *p* < 0.05, **** = *p* < 0.0001, two way ANOVA.

Since we validated this model for the study of RRVD, we chose to investigate the role of macrophages. These cells are known to play a central role in the pathogenesis of arthritogenic alphaviral disease [37]. To study their role in real time in vivo and evaluate an anti-inflammatory therapeutic strategy, we treated mice with Bindarit, an anti-inflammatory small molecule that acts through inhibition of the monocytes chemoattractant proteins (MCPs). We infected mice 6 h after the first administration of the drug with a dose of 10^3^ pfu of RRV-NLuc and monitored the viral dissemination across five days. We observed a significant reduction in the viremia at 2 dpi in the Bindarit treated group when compared to the vehicle treated group (*p* < 0.0001, two way ANOVA; Figure 4a). When measuring the in vivo bioluminescence in the infected animals, we observed no significant difference between the Bindarit and vehicle treated animals in the inoculated feet suggesting no local effect of macrophage inhibition at the site of inoculation. To observe the potential effect of Bindarit treatment on dissemination from the foot to the rest of the body, we quantified the bioluminescence in the whole mouse body (Supplementary Figure S2). At 1 and 3 dpi, no significant difference was observed between the treated and control groups. However, at 5 dpi, the treated mice exhibited a lower bioluminescent signal then the control group (*p* = 0.03, two way ANOVA). When examining a distant site, like the cardiac region, we observed the same trend with no difference upon treatment at 1 and 3 dpi and a lower signal at 5 dpi (*p* = 0.02, two way ANOVA; Figure 4 and Figure S2). To further investigate the consequences of the Bindarit treatment on the viral dissemination, animals were sacrificed on 6 dpi and different organs were collected for ex vivo evaluation of luciferase activity and viral load. First, we measured the bioluminescent signal and did not observe any reduction in signal in the ankle joints upon treatment (Figure 4c). However, in remote joints, such as the knee and hip joints, we observed a reduction in the luciferase activity in the treated group when compared to the vehicle treated group (*p* < 0.05, two way ANOVA; Figure 4c). This trend was also observed in the muscles; the reduction in viral burden was more pronounced in locations further from the inoculation site, such as the masseters (Figure 4d and Figure S2d).

### 3.4. Investigation of Long-Term Viral Replication

**Figure 5:**
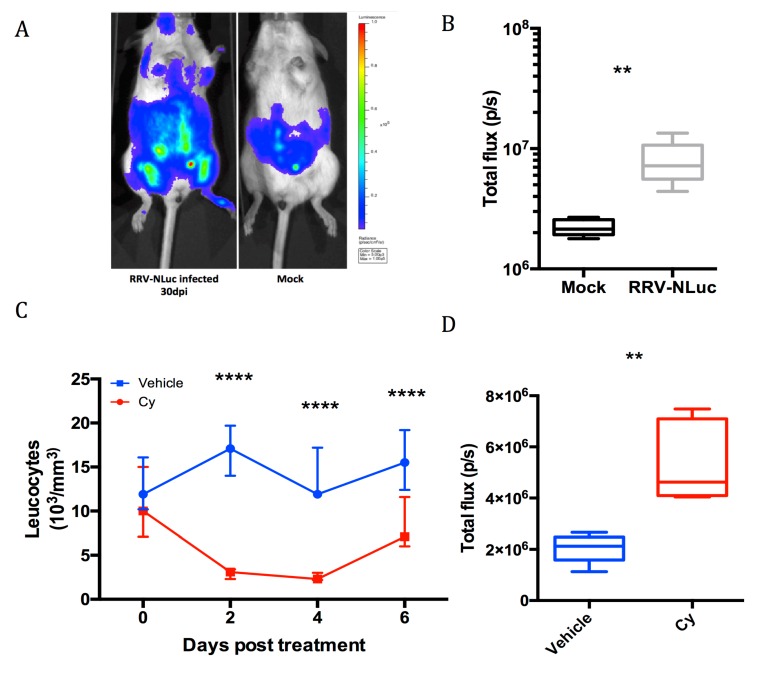
RRV chronic infection is revealed by luminescence of RRV-NLuc and cyclophosphamide immunosuppression. Groups of five mice were infected with 10^3^ pfu RRV-NLuc and monitored for bioluminescence from 30 dpi. (**A**) In vivo imaging of RRV-NLuc infected (left) and mock inoculated (right) mice at 30 dpi; (**B**) total flux counts on the whole body of RRV-NLuc infected and mock-inoculated mice at 30 dpi. The infected group exhibits a significant bioluminescent signal (** = *p* < 0,01 Mann-Whitney); (**C**) leucocytes counts after Cy immunosuppressive (red) treatment or (blue) vehicle, median and range are represented (**** = *p* < 0.0001 two way ANOVA) and (**D**) increase in the total flux counts at 6 dpi after Cy treatment (** = *p* < 0.01, Mann-Whitney).

To test the possibility of observing viral replication in the post-acute and chronic phases with our imaging model, we performed live imaging of the infected mice after resolution of the acute symptoms (30 dpi, Figure 5a). Despite the absence of detectable viremia, we detected bioluminescent signal that indicates viral replication until 30 dpi in the mice inoculated with RRV-NLuc (Figure 5a,b). The signal at this time point (mean = 8.10^6^ p/s) was lower than the signal observed during the acute phase (mean signal at 5 dpi = 2.10^9^ p/s, *p* < 0.0001, Mann-Whitney) but remained significantly higher than the non-infected control (mean control signal = 2, 2.10^6^ p/s; *p* = 0.002, Mann-Whitney; Figure 5a).

To show that replicative virus generated the observed signal, we induced immunosuppression in those mice, expecting this to lead to an enhancement of viral replication. At 30 dpi, we subjected mice to a cyclophosphamide (Cy) treatment using a strategy previously described for reactivation of latent herpesvirus infection [34]. To monitor the immunosuppression and viral replication, we performed peripheral blood leucocytes counts (Figure 5c and Figure S3) and in vivo imaging before and during the treatment. From two to six days post-treatment, we observed a significant decrease in the number of circulating leucocytes (*p* < 0.0001, two way ANOVA) confirming the effectiveness of the immunosuppressive strategy (Figure 5c). As expected, no difference in bioluminescent signal was observed outside of the immunosuppression period. However, at 36 dpi, after the peak of immunosuppression (day four to six post-treatment), we observed an enhancement of the bioluminescent signal in the treated group compared to the mock (*p* = 0.007, Mann-Whitney) (Figure 5d)**.**

## 4. Discussion

Recombinant viruses expressing luciferase genes are powerful tools for viral pathogenesis studies and offer numerous advantages. When implemented for in vivo studies, they allow the use of fewer animals and a faster and easier data collection compared to traditional methods. Moreover, in vivo imaging reduces the possibility of missing biological events happening in non-investigated locations or time-points. Observing the course of the infection with bioluminescence offers other advantages like following the progression of the disease in the same animal through the course of the infection. This strategy has already proven its efficacy and allowed new insights in the study of viral pathogenesis, discovery of novel sites of viral replication and assessment of antiviral strategies and vaccines for viruses as different as herpes and influenza [16,18,38,39]. However, the use of recombinant viruses can have some limitations due to the genetic instability of the insertion or to in vivo attenuation especially among RNA viruses with limited coding capacities [22,38]. Previously designed recombinant alphaviruses expressing luciferases have exhibited some of these limitations. Phillips and colleagues used recombinant encephalitic alphaviruses expressing Firefly luciferase and noticed in vivo attenuation due to the size of the inserted gene (1.6 Kb) [18]. Due to the small size of the NanoLuc (0.5 Kb), our construct RRV-NLuc exhibited a high stability and near-native replication kinetics in vitro. Moreover, the insertion of the NanoLuc in the non-structural region of the viral genome allowed early expression of the reporter protein making this new recombinant virus a good candidate for in vivo studies. This strategy has been previously shown effective with other related alphaviruses as demonstrated by Sun and colleagues [22]. However, different mechanisms underlie the pathogenesis of alphaviral arthritis, and the few in vivo imaging studies published up to date focused on CHIKV, leaving the physiopathological mechanisms of RRVD unexplored [40].

Here we show that adult immunocompetent mice infected with RRV-NLuc showed similar patterns of weight gain, swelling of the inoculated foot and viremia compared to the wild-type virus proving the relevance of using our new recombinant virus for RRVD pathogenesis studies. Moreover, the brightness of this novel luciferase and the use of an albino mice strain reduced the impact of light absorbance by tissues, skin and fur and allowed highly sensitive detection of viral replication in vivo and the detection of a strong bioluminescent signal in the inoculated limb and in distant replication sites (Supplementary Video S4). RRV-NLuc exhibited a dissemination pattern similar to the native RRV T48 strain observed in our laboratory and by other teams [10,11] The highest intensity of signal was observed between 5 and 7 dpi concomitantly to the peak of disease severity and reflected replication in the targeted tissues. RRV-NLuc exhibited muscular and articular tropism, and thus mimics remarkably the in vivo characteristics of the parental strain. In addition, the NanoLuc insertion appeared to be particularly stable in vivo after several days of infection as shown by Sanger sequencing.

To our knowledge, this is the first non-invasive longitudinal follow-up with a recombinant RRV producing a high and long-lasting bioluminescent signal visible beyond the inoculated limb, at variance with other studies involving recombinant arthritogenic alphaviruses [20,21,22].

To fully explore the potential of this RRV in vivo imaging model, we investigated the role of monocytes and macrophages in viral dissemination during the infection. Indeed, if the central role of macrophages in the pathogenesis of arthritogenic alphaviral disease has been clearly established, their role in propagating the infection was not thoroughly studied [37]. Many studies have shown infiltration of monocytic cells in the affected joints and muscles after CHIKV or RRV infection [41,42] and amelioration of the disease outcome in murine models after the inhibition of their recruitment [35,43,44]. With RRV-NLuc, we were able to broaden these observations and evaluate dissemination in the whole mouse. After inhibition of monocytes recruitment, we observed a reduction in viral replication in joints and muscles distant from the inoculation site indicating a role of macrophages in propagating the virus, in addition to their role in the development of the disease [35,44]. Moreover, these findings were corroborated by the study of viremia and bioluminescence showing that this novel imaging model is also adequate for evaluating in vivo intervention strategies, like anti-inflammatory treatments, and could thus be deployed for vaccine or antivirals testing.

Another striking characteristic of alphaviruses is their capability to induce prolonged chronic symptoms. This feature of the disease is particularly difficult to observe and study in animal models, therefore, its aetiology is still discussed with two main hypotheses: Persistence of the replicative virus during the late stages; or chronic pro-inflammatory environment after viral clearance [28,29]. Here we show that mice inoculated with a low dose of RRV-NLuc (10^3^ pfu) exhibit long-term bioluminescent signal after the resolution of the acute phase. These data are supported by previous findings showing RRV persistent infections of several cell types in vitro and presence of viral RNA in patients with chronic RRVD [23,24,25,45]. Moreover, only mice receiving low infectious doses exhibited long lasting bioluminescent signal suggesting a role of the inoculated dose in the development of persistent infections. It is known that different inoculation doses may lead to different outcomes depending on the virus and hosts factors [46]. In our model, lower viral inputs may promote lower immune response and, therefore, viral persistence. Low inoculums are also closer to mosquito inoculated-doses [47], and thus more relevant physiologically. Persistent bioluminescent signal was previously observed with other alphaviruses, Teo and colleagues reported a long-term bioluminescent signal in mice infected with CHIKV expressing luciferase, however this signal was low, restricted to the inoculated foot and did only allow observation and no deeper investigations [21]. Here, the localization of the bioluminescent signal during the chronic phase is not limited to the inoculated limb and suggests persistence of the virus in musculoskeletal tissues and associated lymphoid tissues; however, further investigations are required to determine the precise localization of the viral persistence. In support of our observations, persistent infection has already been reported in vitro for RRV in muscle cells and in CHIKV infected patients up to three months in muscles and up to 18 months in joints [23,26,27]. To date, all the chronicity studies showed presence of viral antigens and/or viral RNA, with no infectious virus being isolated from either experimental animal models or from patients. The quantity of infectious virus possibly present during the late stages of infection could be under the detection limit of current techniques. To demonstrate the association between the bioluminescent signal and replicative virus after the resolution of the acute phase, we subjected mice to an immunosuppressive treatment using a strategy previously described for reactivation of latent viral infections [34]; thus releasing persistent viruses from the control of the adaptive immune response [48,49]. In all the treated mice, we observed an effective immunosuppression and observed an enhanced bioluminescent signal after the peak of immunosuppression. This transient signal suggests a short burst in viral replication triggered by the cyclophosphamide treatment similar to relapses observed in vitro in RRV-persistently infected macrophages [25]. Moreover, the pattern of transient reactivation reported here is similar to the flaring chronic manifestations associated with RRV and CHIKV diseases in humans [8,27]. The data shown here weigh in favor of the viral persistence and reactivation hypothesis; however, further investigations are needed, especially regarding the isolation of infectious virus during the chronic stage and the confirmation of a causal link to the symptoms observed in humans.

With near-parental replication kinetics, a stable construct and a strong bioluminescent signal, RRV-NLuc provides a non-invasive real-time assessment of viral replication and dissemination, which will prove to be an invaluable tool for subsequent pathogenesis studies and the evaluation of therapeutic strategies.

## Supplementary Materials

The following are available online at https://www.mdpi.com/1999-4915/11/7/584/s1, Figure S1: Ex vivo Bioluminescence imaging, Figure S2: Bioluminescence monitoring after Bindarit treatment, Figure S3: Immunosuppression after cyclophosphamide treatment, Video S4: 3D tomography of RRV-NLuc bioluminescence.
