# Supplementary material for: Bioluminescent Ross River Virus Allows Live Monitoring of Acute and Long-Term Alphaviral Infection by In Vivo Imaging
Source: Viruses. 2019 Jun 27;11(7):584. doi: 10.3390/v11070584 (PMC6669695; doi:10.3390/v11070584)
Supplement: Supplementary file 1 [file viruses-11-00584-s001.zip › Caption supplementary material.docx]

**Supplementary Materials**

**S1 Figure: *Ex vivo* Bioluminescence imaging**

Mice inoculated with 10^3^ pfu of RRV-NLuc, were injected intraperitoneally with furimazine, sacrificed and dissected at 6 dpi. Selected organs were screened for bioluminescence *ex vivo* using an IVIS Spectrum Camera. Bioluminescent signal was observed in masseter and cardiac muscles. Images are not to scale.

**S2 Figure: Bioluminescence monitoring after Bindarit treatment**

Mice were treated with Bindarit (grey) or vehicle treated (black) 6 hours prior to infection with 10^3^ pfu of RRV-NLuc. Bioluminescence was monitored for 5 days and quantified for the indicated anatomical regions. Data were pooled from two independent experiments (n=8). A. Inoculated foot; B. Whole body; C. Cardiac region; (* = p<0.05, two way ANOVA); D. *Ex vivo* luciferase activity levels in muscles at 6 dpi (n=5)

**S3 Figure: Immunosuppression after cyclophosphamide treatment**

Leucocytes monitoring in peripheral blood after Cy immunosuppressive treatment (red) or vehicle (blue). A. Leucocytes; B. Lymphocytes; C. Monocytes; D. Granulocytes.

**S4 Video: 3D tomography of RRV-NLuc bioluminescence**

Mice were infected with RRV-NLuc and imaged at 8 dpi. The 3D reconstruction of the bioluminescent in the abdomen, thorax and head is shown on the video. The bioluminescent signal present in the limbs is not visible due to software constraint.
