# Supplementary material for: Bioluminescent Ross River Virus Allows Live Monitoring of Acute and Long-Term Alphaviral Infection by In Vivo Imaging
Source: Viruses. 2019 Jun 27;11(7):584. doi: 10.3390/v11070584 (PMC6669695; doi:10.3390/v11070584)
Supplement: Supplementary file 1 [file viruses-11-00584-s001.zip › S5-Fig.pptx]

## Slide 1
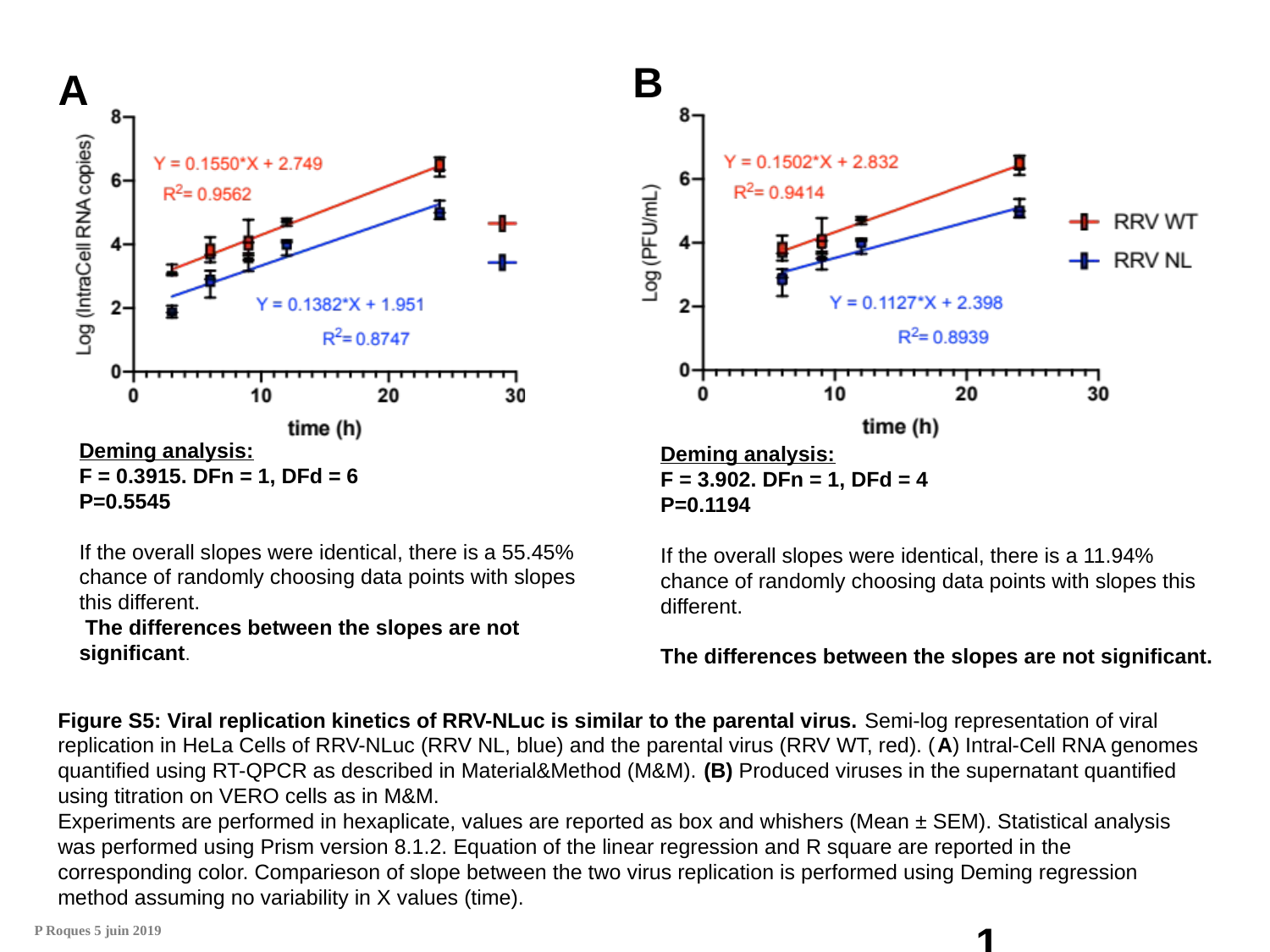

B
A
Deming analysis:
F = 0.3915. DFn = 1, DFd = 6
P=0.5545
If the overall slopes were identical, there is a 55.45% chance of randomly choosing data points with slopes this different.
 The differences between the slopes are not significant.
Deming analysis:
F = 3.902. DFn = 1, DFd = 4
P=0.1194
If the overall slopes were identical, there is a 11.94% chance of randomly choosing data points with slopes this different.
The differences between the slopes are not significant.
Figure S5: Viral replication kinetics of RRV-NLuc is similar to the parental virus. Semi-log representation of viral replication in HeLa Cells of RRV-NLuc (RRV NL, blue) and the parental virus (RRV WT, red). (A) Intral-Cell RNA genomes quantified using RT-QPCR as described in Material&Method (M&M). (B) Produced viruses in the supernatant quantified using titration on VERO cells as in M&M.
Experiments are performed in hexaplicate, values are reported as box and whishers (Mean ± SEM). Statistical analysis was performed using Prism version 8.1.2. Equation of the linear regression and R square are reported in the corresponding color. Comparieson of slope between the two virus replication is performed using Deming regression method assuming no variability in X values (time).
0
