# Supplementary figures and images for: Bioluminescent Ross River Virus Allows Live Monitoring of Acute and Long-Term Alphaviral Infection by In Vivo Imaging
Source: Viruses. 2019 Jun 27;11(7):584. doi: 10.3390/v11070584 (PMC6669695; doi:10.3390/v11070584)

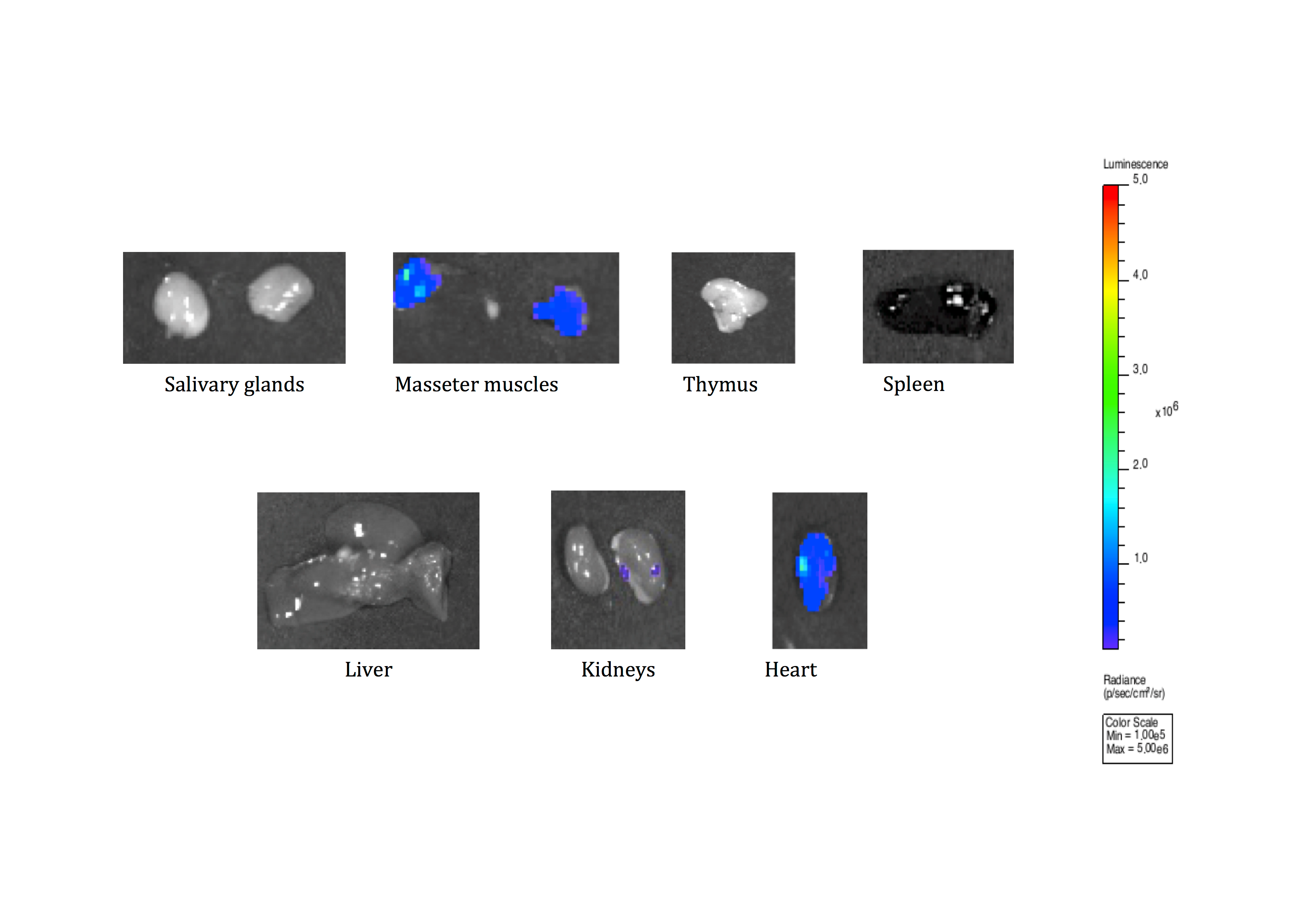

Supplement: Supplementary file 1 [file viruses-11-00584-s001.zip › S1_Fig.tiff]

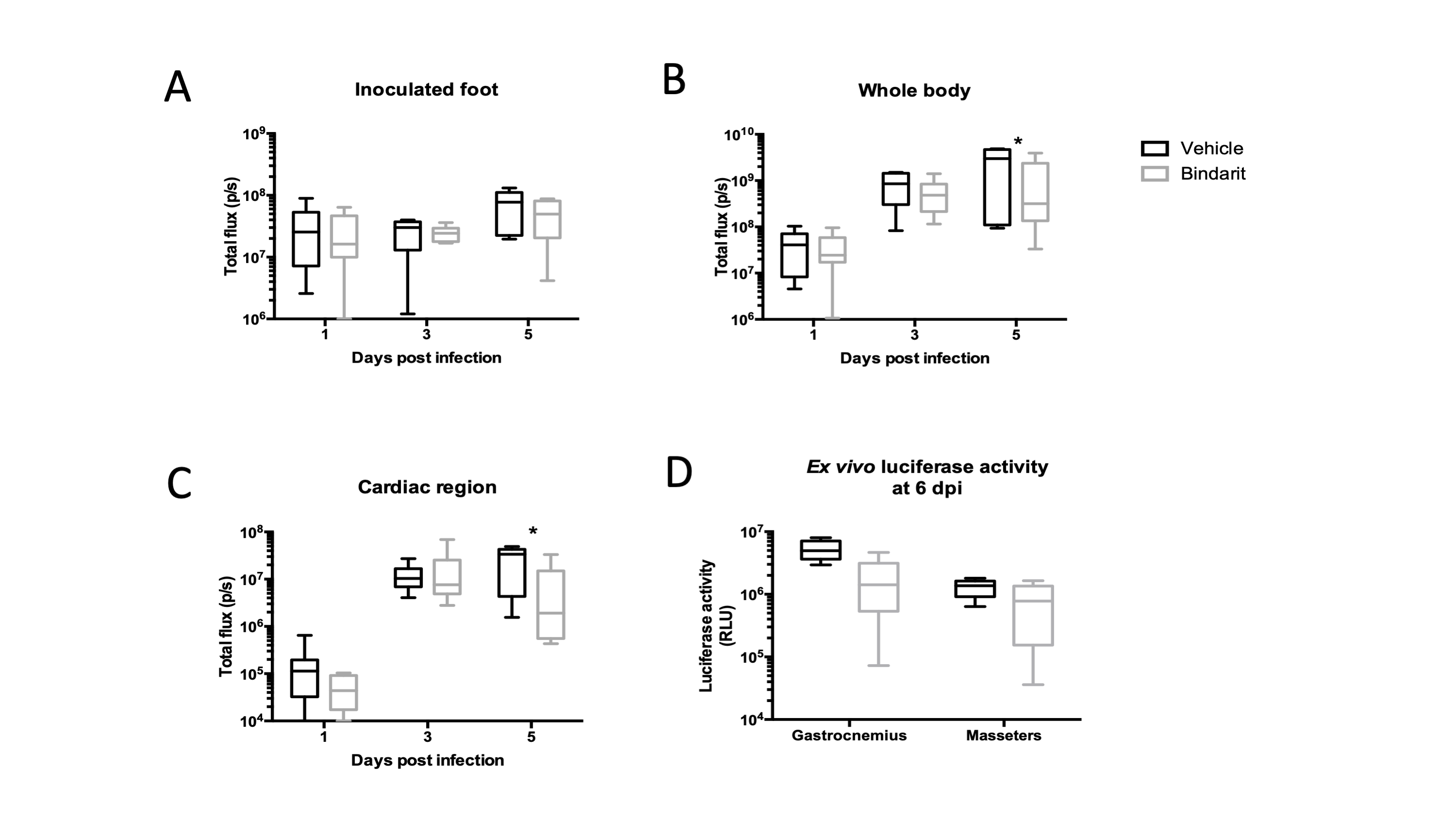

Supplement: Supplementary file 1 [file viruses-11-00584-s001.zip › S2_Fig.tiff]

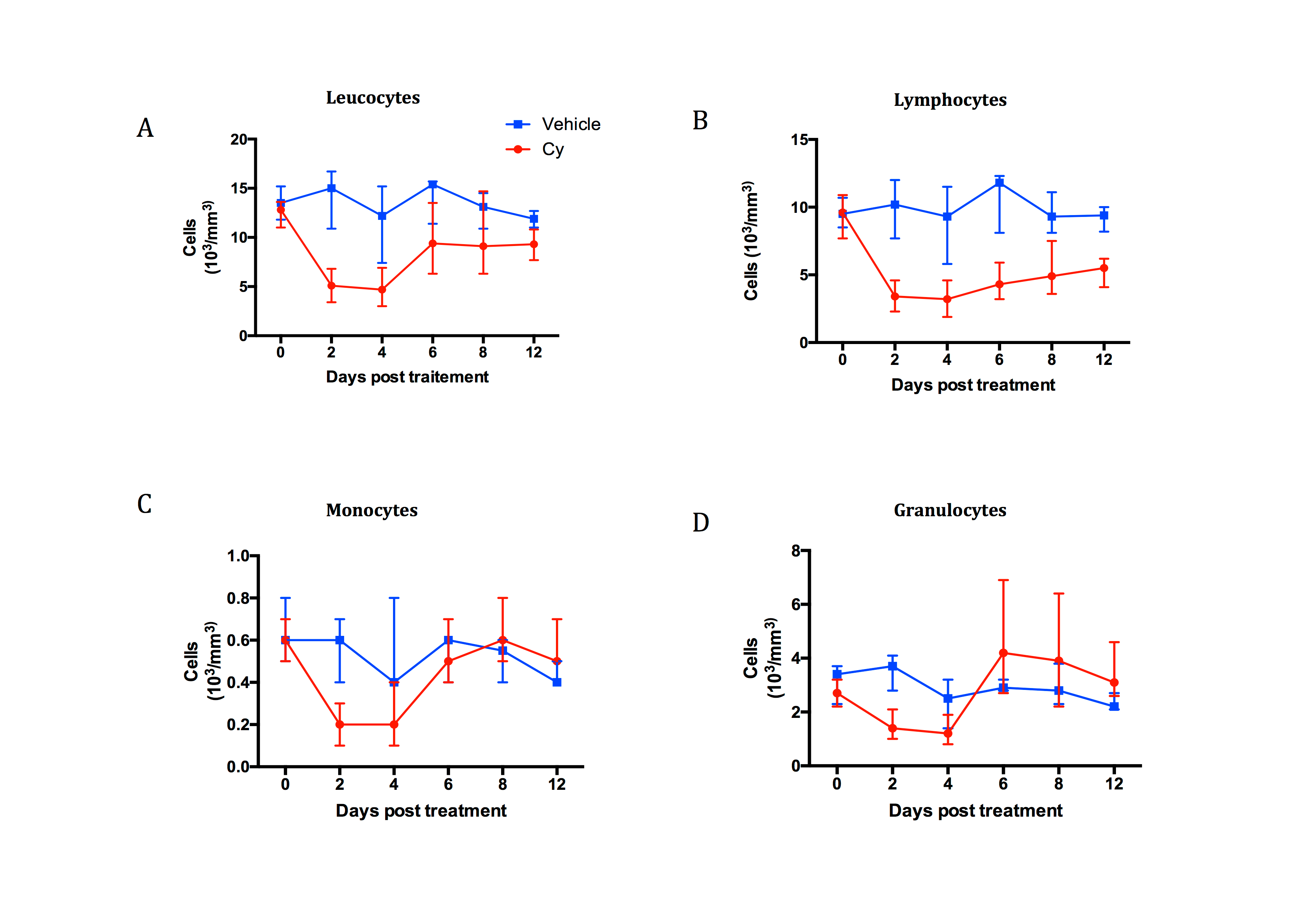

Supplement: Supplementary file 1 [file viruses-11-00584-s001.zip › S3_Fig.tiff]
